# Synergistic antibacterial effect of co-administering adipose-derived mesenchymal stromal cells and *Ophiophagus hannah*l-amino acid oxidase in a mouse model of methicillin-resistant *Staphylococcus aureus*-infected wounds

**DOI:** 10.1186/s13287-016-0457-2

**Published:** 2017-01-23

**Authors:** Yee Yik Mot, Iekhsan Othman, Syed Hassan Sharifah

**Affiliations:** Jeffrey Cheah School of Medicine and Health Sciences and Tropical Medicine and Biology, Infectious Diseases and Health, Monash University Malaysia, Jalan Lagoon Selatan, Subang Jaya, 47500 Selangor Malaysia

**Keywords:** Mesenchymal stromal cells, *Ophiophagus hannah*l-amino acid oxidase, Synergistic antibacterial activity

## Abstract

**Background:**

Mesenchymal stromal cells (MSCs) and *Ophiophagus hannah* l-amino acid oxidase (Oh-LAAO) have been reported to exhibit antimicrobial activity against methicillin-resistant *Staphylococcus aureus* (MRSA). Published data have indicated that synergistic antibacterial effects could be achieved by co-administration of two or more antimicrobial agents. However, this hypothesis has not been proven in a cell- and protein-based combination. In this study, we investigate if co-administration of adipose-derived MSCs and Oh-LAAO into a mouse model of MRSA-infected wounds would be able to result in a synergistic antibacterial effect.

**Methods:**

MSCs and Oh-LAAO were isolated and characterized by standard methodologies. The effects of the experimental therapies were evaluated in C57/BL6 mice. The animal study groups consisted of full-thickness uninfected and MRSA-infected wound models which received Oh-LAAO, MSCs, or both. Oh-LAAO was administered directly on the wound while MSCs were delivered via intradermal injections. The animals were housed individually with wound measurements taken on days 0, 3, and 7. Histological analyses and bacterial enumeration were performed on wound biopsies to determine the efficacy of each treatment.

**Results:**

Immunophenotyping and differentiation assays conducted on isolated MSCs indicated expression of standard cell surface markers and plasticity which corresponds to published data. Characterization of Oh-LAAO by proteomics, enzymatic, and antibacterial assays confirmed the identity, purity, and functionality of the enzyme prior to use in our subsequent studies. Individual treatments with MSCs and Oh-LAAO in the infected model resulted in reduction of MRSA load by one order of magnitude to the approximate range of 6 log_10_ colony-forming units (CFU) compared to untreated controls (7.3 log_10_ CFU). Similar wound healing and improvements in histological parameters were observed between the two groups. Co-administration of MSCs and Oh-LAAO reduced bacterial burden by approximately two orders of magnitude to 5.1 log_10_ CFU. Wound closure measurements and histology analysis of biopsies obtained from the combinational therapy group indicated significant enhancement in the wound healing process compared to all other groups.

**Conclusions:**

We demonstrated that co-administration of MSCs and Oh-LAAO into a mouse model of MRSA-infected wounds exhibited a synergistic antibacterial effect which significantly reduced the bacterial count and accelerated the wound healing process.

**Electronic supplementary material:**

The online version of this article (doi:10.1186/s13287-016-0457-2) contains supplementary material, which is available to authorized users.

## Background

Bacterial infection has been identified as a major causative factor in the impairment of wound healing [1]. Methicillin-resistant *Staphylococcus aureus* (MRSA) is a widespread staphylococcal strain responsible for many local and systemic infections and is known to rapidly develop resistance towards commonly prescribed antibiotics. Consequently, the observed prevalence and adaptation of MRSA together with the recent appearance of vancomycin-resistant strains has resulted in an increasing spectrum of untreatable staphylococcal infections [2]. Therefore, novel approaches to treat MRSA infections and counter the increasing problem of antimicrobial resistance (AMR) are urgently required.

Bioprospecting of therapeutic compounds from snake venoms has identified a myriad of proteins which exhibit antimicrobial activities. l-amino acid oxidase isolated from *Ophiophagus hannah* (Oh-LAAO) has been demonstrated to be more efficacious in its antimicrobial activity against many strains of Gram-positive bacteria commonly associated with cutaneous wounds compared to routinely used antibiotics [3]. The antimicrobial activity of Oh-LAAO on MRSA has also been previously demonstrated [4], as has properties of thermal stability and retention of activity under alkaline conditions [5].

Mesenchymal stromal cells (MSCs) have been explored for cutaneous wound healing as they demonstrate a high regenerative capacity and in vivo immunomodulatory effects. Ex vivo studies with MSCs have demonstrated their active participation in the wound healing process via a multitude of mechanisms including angiogenesis, differentiation, secretion of paracrine factors, promotion of cell migration, re-epithelialization, restoration of sebaceous glands and hair follicles, increased collagen deposition, enhanced tissue granulation, a decrease in inflammatory response, and local cell engraftment [6–9]. In addition, administration of MSCs in infected models has been reported to result in beneficial outcomes including eliciting anti-inflammatory and anti-apoptotic responses, neoangiogenesis, immunomodulation, and activation of resident stem cells [10]. Furthermore, MSCs have also been demonstrated to secrete the antimicrobial peptide (AMP) LL-37 when challenged with *Escherichia coli* in an in vivo mouse pneumonia model [11]. In terms of skin infections, MSCs introduced by intravenous tail injection into subcutaneous MRSA-infected rats were able to reduce the bacterial load in a dose-dependent manner [12].

Published data have indicated that synergistic antibacterial effects could be achieved by co-administration of two or more antimicrobial agents, which may consists of antibiotics [13], proteins [14], or essential oils [15]. These combinations have been shown to enhance antibacterial activity towards resistant and nonresistant bacterial strains. It is suggested that compounds which target the cell wall or cell membrane are likely to synergize the effects of accompanying drugs as a result of increased permeability [13]. However, this hypothesis has not been proven in a cell- and protein-based combination. Here, we describe, for the first time, studies of the synergistic antibacterial effect of co-administering MSCs and Oh-LAAO in a mouse model of MRSA-infected cutaneous wounds. We found that there were significant synergistic antibacterial activities when MSCs and Oh-LAAO were co-administered in infected models which led to substantial bacterial load reduction and enhanced improvements in the wound healing process.

## Methods

### Isolation and characterization of MSCs

MSCs were isolated as previously described [16]. Briefly, adipose tissues were collected from subcutaneous sites of male C57/BL6 mice and digested with 1 mg/ml collagenase IA for 1 h at 37 °C in a shaking incubator. The released cells were separated by centrifugation and erythrocytes were removed from the sample using the BD Pharm Lyse Lysing Buffer. The cells were then resuspended in StemXVivo™ media and maintained in a 37 °C humidified incubator with 5% carbon dioxide. The confluent cultures were passaged and amplified until a morphologically homogenous cell population was achieved.

### Characterization of mouse adipose-derived MSCs

Standard characterization of MSCs was performed by immunophenotyping of cell surface markers and differentiation into adipocytes, osteocytes, and chondrocytes. Immunophenotyping was performed using the Mouse Mesenchymal Stem Cell Marker Antibody Panel (R&D Systems). Flow cytometry analysis was performed using the FACSVantage™ system and CellQuest software. Differentiation of the MSCs into adipocytes, osteocytes, and chondrocytes was performed using the Mouse Mesenchymal Stem Cell Functional Identification Kit (R&D Systems) [17].

### Purified Oh-LAAO

The purified Oh-LAAO used in our study was a kind gift from Sugita Kunalan, Monash University, Malaysia. Characterization of the protein was performed by sodium dodecyl sulfate polyacrylamide gel electrophoresis (SDS-PAGE), Western blot, and liquid chromatography-mass spectrometry/mass spectrometry (LCMS/MS). Enzymatic analyses including LAAO activity, cytotoxicity, and antibacterial assays confirmed functionality and a minimum inhibitory concentration (MIC) value similar to published data [4]. Purity of the enzyme was reconfirmed by SDS-PAGE prior to in vivo studies.

### In vivo study of MSC and Oh-LAAO treatment on infected cutaneous wounds

The wound model was created using male C57/BL6 mice aged 6 to 7 weeks old. The animals were maintained in individually ventilated cages under climate-controlled conditions of 12 h light/dark cycles at 22.0 ± 3 °C. Standard rodent chow pellets and water were provided ad libitum. A biopsy punch was used to create a 5-mm full-thickness excisional skin wound on the dorsal midline [18]. The wounds were inoculated with 10^7^ colony-forming units (CFU) MRSA. Five treatment groups were planned: 1) co-administration of MSCs and Oh-LAAO; 2) MSCs only; 3) Oh-LAAO only; 4) Fusidic acid ointment (FAO); and 5) phosphate-buffered saline (PBS) (*n* = 6). A parallel study of the treatment groups was performed on uninfected models. MSCs were administered by intradermal injections consisting of 1 × 10^6^ cells. Oh-LAAO was administered at a concentration of 10 mg/kg body weight. MSCs were introduced by intradermal injection into four sites adjacent to the wound edges. A digital calliper was used to measure the extent of the wound size on day 0, day 3, and day 7 of the observation period.

### Enumeration of MRSA at the wound sites

Enumerations of MRSA on cutaneous samples obtained from the in vivo studies were performed with CHROMagar™ MRSA. Skin biopsies of the wound site were homogenized and dilutions to 10^8^ were made in PBS before spreading onto CHROMagar plates. The inoculated plates were incubated at 37 °C for 24 h and the CFU/ml for each treatment group was determined.

### Histopathological analyses of wound biopsies

Skin biopsy specimens obtained from each in vivo study group were fixed in 10% (v/v) neutral-buffered formalin. The specimens were embedded in paraffin blocks and sliced with a microtome to 5-μm sections for subsequent Masson’s Trichrome and hematoxylin and eosin (H&E) staining. The tissue specimens were examined by microscopy for determination of collagen deposition, angiogenesis, fibroblast proliferation, epithelialization, and tissue granulation.

Histological scores were assigned based on a previously published method [19]. Points allocation were determined as follows: 1–3, absence of granulation tissue and epithelial migration with minimal cell accumulation; 4–6, thin and immature granulation tissues consisting predominantly of inflammatory cells—a limited number of fibroblasts, blood capillaries, and collagen deposition is observed accompanied by minimal epithelial migration; 7–9, moderately thick granulation tissues with cellular accumulation ranging from predominantly inflammatory cells to increasing numbers of fibroblasts and collagen deposition—extensive neovascularization is present accompanied by minimal to moderate epithelium migration; and 10–12, thick, vascular granulation tissues consisting predominantly of fibroblasts accompanied by extensive collagen deposition—epithelium migration has extended to partially to completely covering the wound area.

### Statistical analysis

All experimental data were expressed as mean ± standard deviation (SD). Statistical analyses were performed by one-way analysis of variance (ANOVA) followed by the Tukey HSD test. Values obtained which represented *p* < 0.05 were considered to be statistically significant.

## Results

### Isolated treatment components, MSCs and Oh-LAAO, conform to standard validation assays

MSCs isolated from the adipose tissues were heterogeneous at passage 0 and gradually attained a uniform fibroblastic morphology by passage 3 [16]. The immunophenotyping results indicated the cells were positive for the stromal markers Sca-1, CD106, CD105, CD73, CD29, and CD44, while they were negative for CD45 and CD11b (see Additional file 1: Figure S1) which corresponds to published data [16]. Additionally, a differentiation assay indicated that the isolated cells were able to transdifferentiate into adipocytes, osteocytes, and chondrocytes (see Additional file 1: Figure S2) [20].

SDS-PAGE conducted on purified Oh-LAAO revealed a single band with a molecular weight of approximately 68 kDa (see Additional file 1: Figure S3) which corresponds to data previously reported to be the expected size of Oh-LAAO [4].

### Combinational therapy of MSCs and Oh-LAAO synergistically reduces bacterial load and reverses the wound healing delay caused by infection

**Fig. 1:**
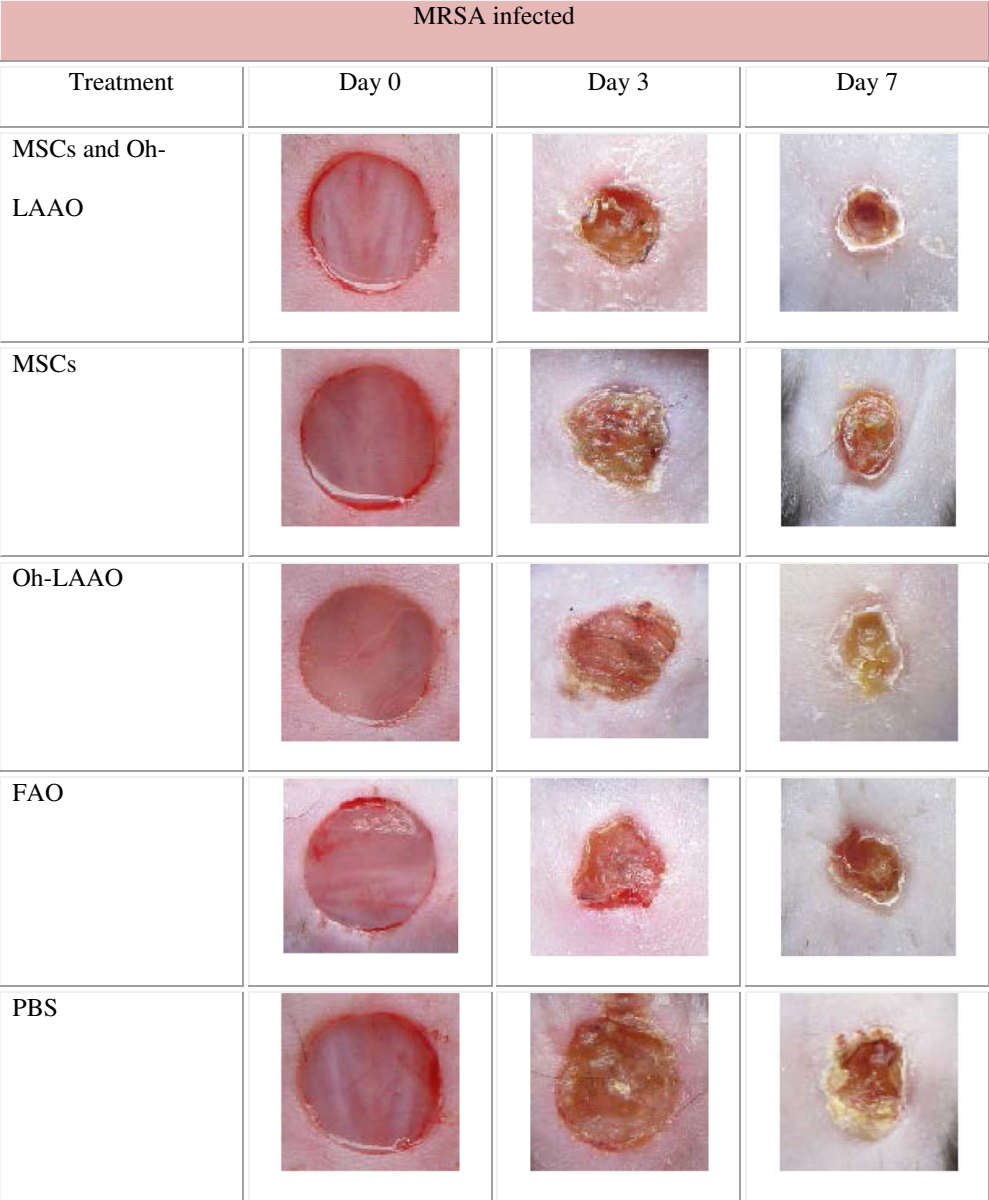
Wound healing rate of the methicillin-resistant *Staphylococcus aureus* (*MRSA*)-infected mouse cutaneous wound model at days 0, 3, and 7. The animals were divided into five study groups: mesenchymal stromal cells (*MSCs*) in combination with *Ophiophagus hannah* l-amino acid oxidase (*Oh-LAAO*), MSCs only, Oh-LAAO only, Fusidic acid ointment (*FAO*), and phosphate-buffered saline (*PBS*)

**Fig. 2:**
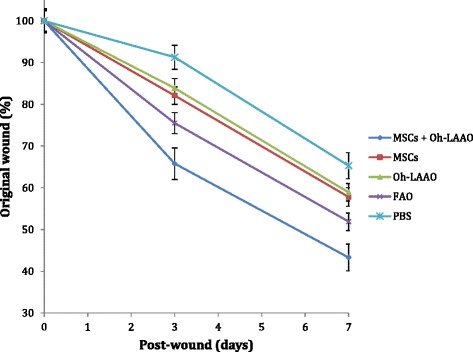
Wound healing rate of MRSA-infected study groups consisting of mesenchymal stromal cells (*MSCs*) in combination with *Ophiophagus hannah* l-amino acid oxidase (*Oh-LAAO*), MSCs only, Oh-LAAO only, Fusidic acid ointment (*FAO*), and phosphate-buffered saline (*PBS*). Measurements were taken on days 0, 3, and 7 post-wounding to assess the efficacy of each treatment

**Fig. 3:**
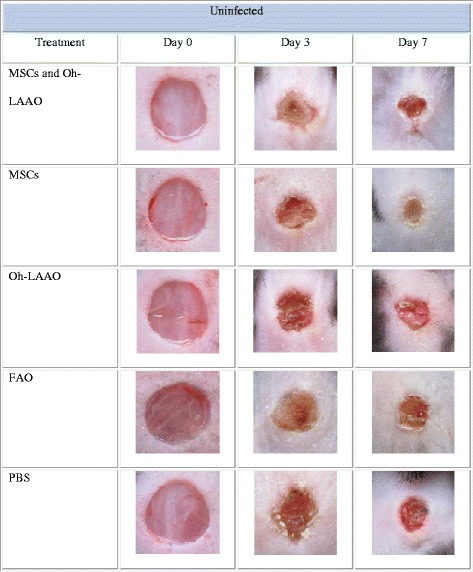
Wound healing rate of the uninfected mouse cutaneous wound model at days 0, 3, and 7. The animals were divided into five study groups: mesenchymal stromal cells (*MSCs*) in combination with *Ophiophagus hannah* l-amino acid oxidase (*Oh-LAAO*), MSCs only, Oh-LAAO only, Fusidic acid ointment (*FAO*), and phosphate-buffered saline (*PBS*)

**Fig. 4:**
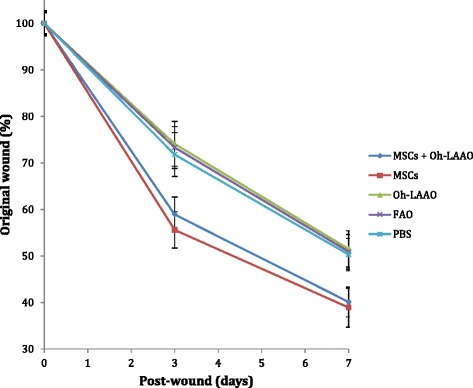
Wound healing rate of uninfected study groups consisting of mesenchymal stromal cells (*MSCs*) in combination with *Ophiophagus hannah* l-amino acid oxidase (*Oh-LAAO*), MSCs only, Oh-LAAO only, Fusidic acid ointment (*FAO*), and phosphate-buffered saline (*PBS*). Measurements were taken on days 0, 3, and 7 post-wounding to assess the in vivo toxicity of each treatment

In the MRSA-infected groups, the highest healing rate was observed for the combinational therapy of MSCs and Oh-LAAO at both day 3 and day 7. This was followed by the FAO treated group. The MSC and Oh-LAAO individual treatment groups healed at similar rates without any significant differences observed. The PBS group was the slowest to heal (Figs. 1 and 2). In the uninfected groups, the highest healing rate was observed for the MSC individual treatment group and combinational therapy of MSCs and Oh-LAAO at both day 3 and day 7. This was followed by the FAO, Oh-LAAO, and PBS groups, which healed at similar rates without any significant differences observed (Figs. 3 and 4).

**Fig. 5:**
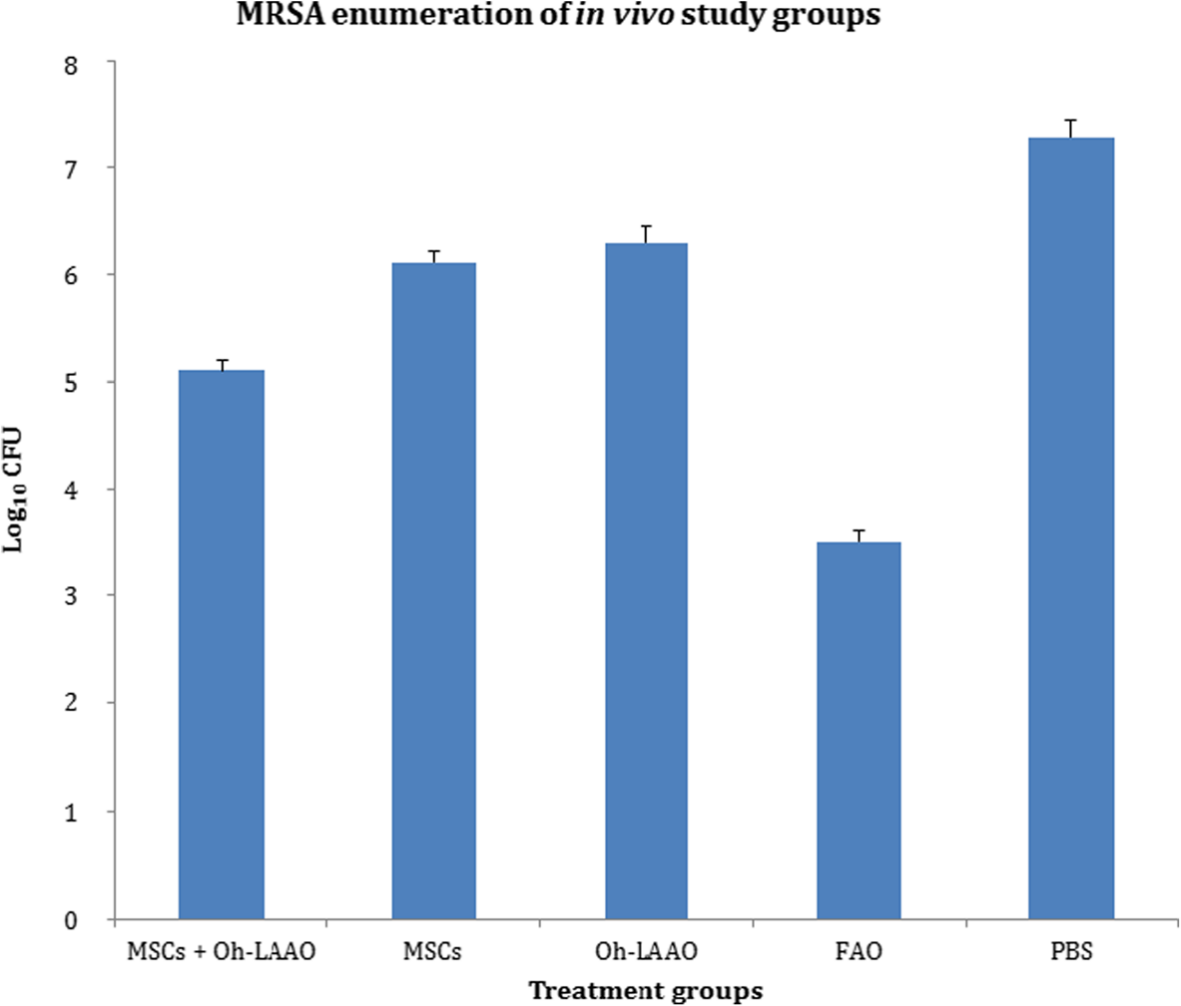
Wound biopsies of methicillin-resistant *Staphylococcus aureus* (*MRSA*)-infected study groups were homogenized and plated on CHROMagar for enumeration of mauve colonies. All treatment groups demonstrated reduction of bacterial load compared to the untreated (phosphate-buffered saline; *PBS*) control. The most significant reduction was observed in the standard antibiotic group (Fusidic acid ointment; *FAO*). In the experimental therapy groups, the mesenchymal stromal cells (*MSCs*) and *Ophiophagus hannah* l-amino acid oxidase (*Oh-LAAO*) combinational treatment resulted in the highest reduction of MRSA count, by approximately two orders of magnitude. MRSA enumeration of the study groups with individual treatment of MSCs and Oh-LAAO indicated a reduction of one order of magnitude compared to the untreated control

In the MRSA enumeration study, treatment with a standard antibiotic (FAO) resulted in the highest bacterial reduction to approximately 3.5 log_10_ CFU. This was followed by the combinational therapy, with a reduction of approximately two orders of magnitude to 5.1 log_10_ CFU. Treatment with MSCs resulted in 6.1 log_10_ CFU of surviving MRSA while the Oh-LAAO treated group reduced bacterial count to 6.3 log_10_ CFU (Fig. 5).

### Treated groups indicated improvements in histopathological assessments

**Fig. 6:**
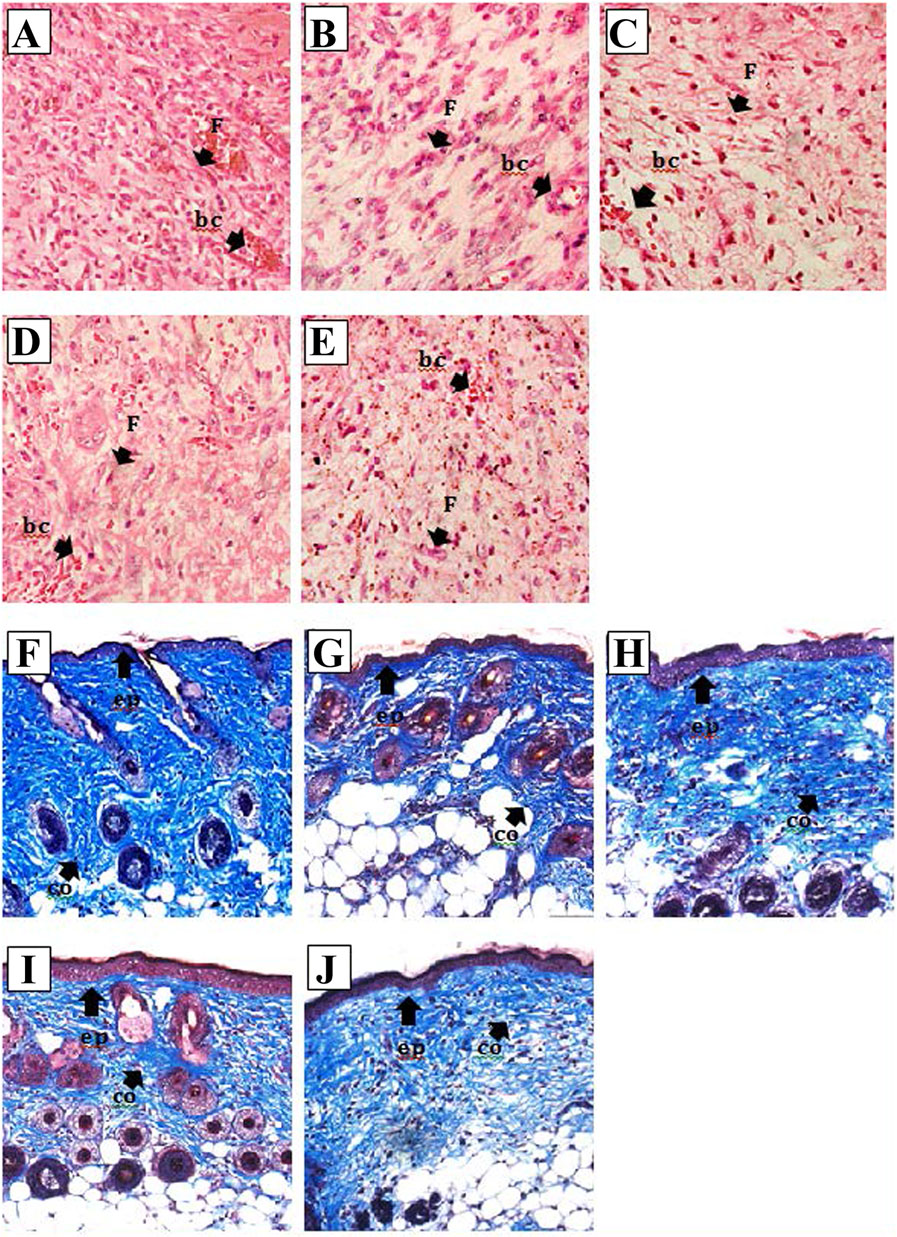
Histological evaluation of MRSA-infected wound biopsies stained with H&E (**a**–**e**) and Masson’s trichrome (**f**–**j**). Treatments were performed with combinational therapy of MSCs and Oh-LAAO (**a**, **f**), MSCs (**b**, **g**), Oh-LAAO (**c**, **h**), FAO (**d**, **i**), and PBS (**e**, **j**). *bc* blood capillaries, *co* collagen fibers, *ep* epithelialization, *F* fibroblast

Histological analyses were performed on tissue samples obtained at the end of the 7 days in vivo study duration. In the MRSA-infected groups, combinational therapy with MSCs and Oh-LAAO demonstrated the most significant improvements in all the assessed parameters (Fig. 6). This was followed by the FAO treated group which presented a comparable amount of collagen accumulation but moderate formation of blood capillaries, fibroblast infiltration, and re-epithelialization. The MSC and Oh-LAAO individual treatment groups demonstrated similar improvements. However, the MSC group exhibited more evident neovascularization compared to the Oh-LAAO group.

**Fig. 7:**
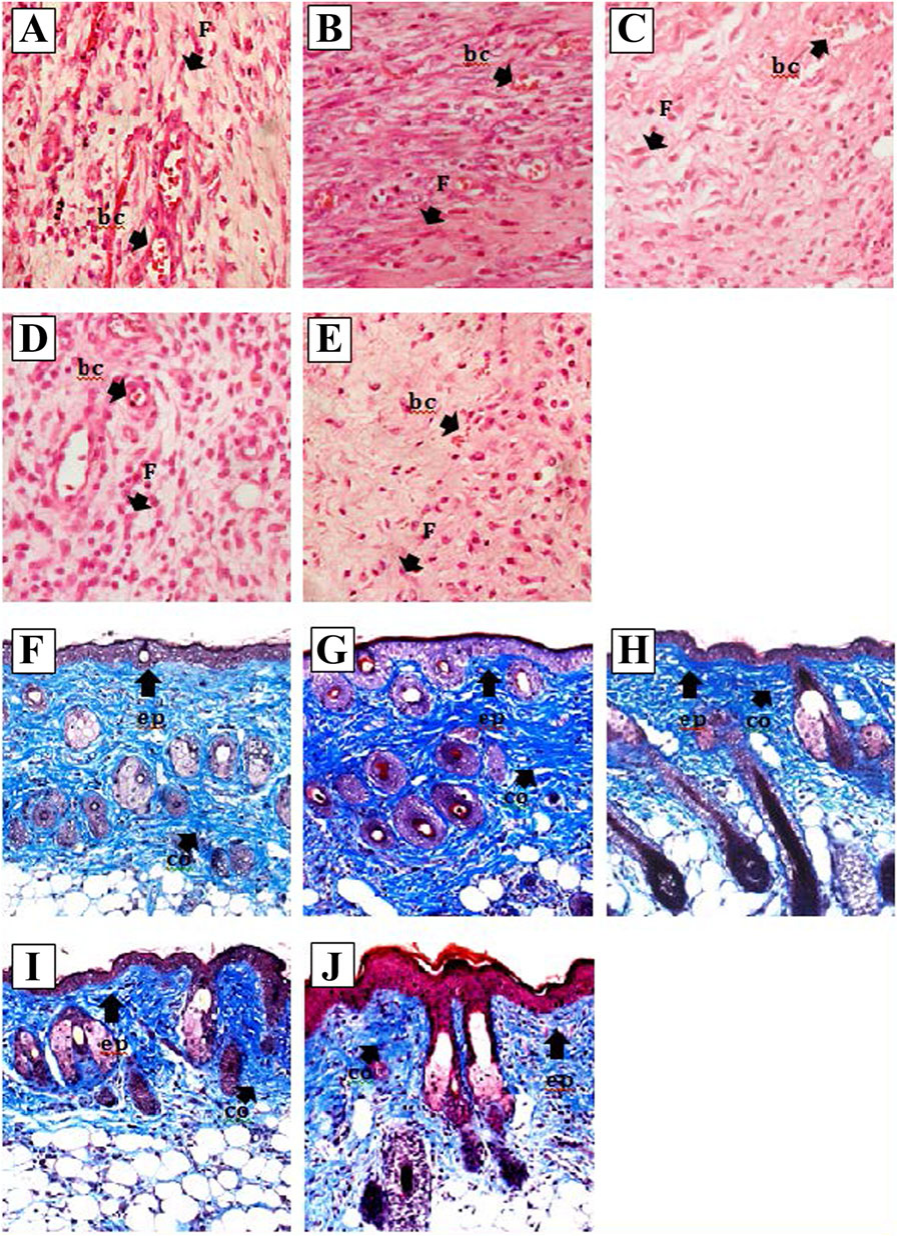
Histological evaluation of uninfected wound biopsies stained with H&E (**a**–**e**) and Masson’s trichrome (**f**–**j**). Treatments were performed with combinational therapy of MSCs and Oh-LAAO (**a**, **f**), MSCs (**b**, **g**), Oh-LAAO (**c**, **h**), FAO (**d**, **i**), and PBS (**e**, **j**). *bc* blood capillaries, *co* collagen fibers, *ep* epithelialization, *F* fibroblast

In the uninfected groups, the most significant improvements in the assessed histological parameters were observed in the combinational therapy and MSC treated groups (Fig. 7). The Oh-LAAO, FAO, and PBS groups demonstrated reduced healing compared to those with the inclusion of MSCs. In general, it was evident that the MRSA-infected groups demonstrated reduced improvements in the histological parameters assessed compared to the corresponding uninfected groups, indicating that the MRSA infection produced in our study significantly reduced healing rates of the open wounds. A summary of the histopathological assessment of the wound healing of infected and uninfected animal study groups is provided in Table 1.

**Table 1:** Wound area assessment at days 3 and 7 of in vivo study and histopathological scores of healing parameters performed on biopsy samples sectioned and stained by hematoxylin and eosin and Masson’s trichrome

		% Wound area	
Treatment	Histological score	Day 3	Day 7
MRSA-infected			
MSCs and Oh-LAAO	9.8 ± 0.6	65.8 ± 3.8	43.3 ± 3.2
MSCs	6.1 ± 0.5	82.1 ± 2.1	57.8 ± 2.2
Oh-LAAO	5.7 ± 0.3	83.9 ± 2.3	58.9 ± 2.1
FAO	6.4 ± 0.3	75.5 ± 2.5	51.9 ± 2.1
PBS	3.7 ± 0.4	91.3 ± 2.9	65.3 ± 3.1
Uninfected			
MSCs and Oh-LAAO	11.3 ± 0.5	58.9 ± 3.8	40.1 ± 3.2
MSCs	11.5 ± 0.3	55.6 ± 3.9	38.9 ± 4.2
Oh-LAAO	6.6 ± 0.7	74.1 ± 4.8	51.5 ± 3.9
FAO	6.7 ± 0.5	73.3 ± 4.5	50.9 ± 3.7
PBS	6.9 ± 0.4	71.8 ± 4.7	50.3 ± 3.5

*FAO* Fusidic acid ointment, *MSCs* mesenchymal stromal cells, *Oh-LAAO Ophiophagus hannah* l-amino acid oxidase, *PBS* phosphate-buffered saline

## Discussion

Here we describe the combinational treatment of an antimicrobial protein, Oh-LAAO, and cell replacement therapy with MSCs which exhibit both a regenerative capacity and antibacterial properties in the mitigation of an MRSA-infected cutaneous wound model. Our findings in the in vivo study indicated that individual treatment with Oh-LAAO reduced the inoculated MRSA load by one order of magnitude with no significant signs of toxicity or wound closure delays in both the infected and uninfected models. There was also no observed deterioration in the histological findings and, therefore, Oh-LAAO could be classified as a relatively safe protein drug with the potential to be developed as a therapeutic candidate. To the best of our knowledge, snake venom LAAOs have not been investigated in vivo as an antibacterial agent. Although research into the antimicrobial activity of snake venom LAAOs is abundant [21], much of the work has focused on in vitro research. In a similar animal study, *Crotalus adamanteus* toxin II (CaTx-II) isolated from the venom of *Crotalus adamanteus* was used as an antibacterial agent to investigate its effects on a *Staphylococcus aureus*-infected mouse cutaneous wound model [22]. Comparatively, it was noted that an in vitro antibacterial assay of both Oh-LAAO and CaTx-II indicated similar MIC and minimum bactericidal concentration (MBC) values. However, in the in vivo study it was found that Oh-LAAO exerted a lower antibacterial effect compared to CaTx-II, as demonstrated by the end-point bacterial CFU enumeration. This observation could be partly explained by the size and stability of the isolated enzymes, as it is not uncommon for the half-life of different proteins to vary in vivo based on these characteristics. As degradation of the enzymes progresses, possible structural and functional loss may occur. It is postulated that Oh-LAAO may be more susceptible to the effects of functional loss as it exerts its antibacterial activity by hydrogen peroxide release (requiring an intact catalytic site) which may be compromised by early in vivo degradation of the protein. The mechanism of action of CaTx-II differs from Oh-LAAO and may not be as severely affected by the in vivo microenvironment, and therefore may have prolonged its antibacterial effect.

In comparison to the Oh-LAAO group, the administration of MSCs in the infected model demonstrated a similar reduction in bacterial burden of one order of magnitude accompanied by a comparable wound closure rate. Histological assessments also revealed similar improvements, except that the MSC group indicated higher formation of blood capillaries. These results collectively demonstrated that reduction of bacterial burden led to tandem improvements in the wound healing process. It has previously been demonstrated that human MSCs secrete the AMP LL-37 when challenged with bacteria, which accounts for its direct antimicrobial activity [11]. Therefore, it is postulated that the mouse homolog of LL-37, mCRAMP (mouse cathelicidin-related antimicrobial peptide) [23], was secreted in response to the presence of MRSA in our study leading to the reduction in bacterial count. In addition, previous studies have demonstrated that MSCs may participate in the mitigation of infection by secretion of soluble paracrine factors including interleukin (IL)-10 [24], prostaglandin E_2_ (PGE_2_) [25], tumor necrosis factor (TNF)-α-stimulated gene/protein 6 [26], and IL-6 [27]. A previous study investigating the antibacterial effects of MSCs on a subcutaneous infected rat model indicated a higher reduction of MRSA count compared to our present findings [12]. This observation could be explained by the amount of cells administered into the host animals. In the present investigation, 1 × 10^6^ MSCs were introduced once by intradermal route at the beginning of the study, while the dosing regimen of the previous study was by intravenous injection once a day for 4 days with MSC concentrations varying from 2 × 10^5^ to 2 × 10^7^ cells per animal. Their findings indicated that all cell concentrations used resulted in a lower CFU count compared to our study. As such, improvements to our present research design could be made by incorporation of additional MSC doses and administering higher concentrations of cells.

Furthermore, it was observed that the combinational therapy of MSCs and Oh-LAAO in our study demonstrated greater in vivo antibacterial activity by reducing the MRSA load in the infected model by two orders of magnitude. These results suggest that a synergistic effect was produced by administration of the treatments in combination. To the best of our knowledge, this is the first study to demonstrate that a protein and cell combinational therapy is able to produce synergistic antimicrobial activity. In vitro synergies by antibacterial proteins have been observed by treatment combinations with β-defensins, cathelicidin LL-37, and lysozyme against *Staphylococcus aureus* and *Escherichia coli* [14]. Aside from the reduction in bacterial burden, marked improvements were also observed in the wound closure rate and histological analyses, meaning that the combinational therapy was able to substantially accelerate the wound healing process compared to the individual treatments and controls. Further detailed analysis of the bacterial enumeration results revealed that the combined treatment effectively reduced the MRSA count to the approximate range of 10^5^ CFU, which was demonstrated in our optimization procedures to be insufficient to cause significant delays in wound closure. This finding corroborates with our wound measurement and histological assessment which indicated a marginal difference between the infected and uninfected wounds treated with the combinational therapy. From this finding, it could be postulated that reduction of bacterial burden to below its critical level to manifest infection could negate the delay in wound healing, and that total eradication of bacterial presence in open wounds may not be necessary for the resumption of normal wound closure to take place. This observation is in agreement with published data of human studies which have indicated that a critical level of bacteria of between 10^4^ to 10^6^ CFU must be reached to cause infection [28].

**Fig. 8:**
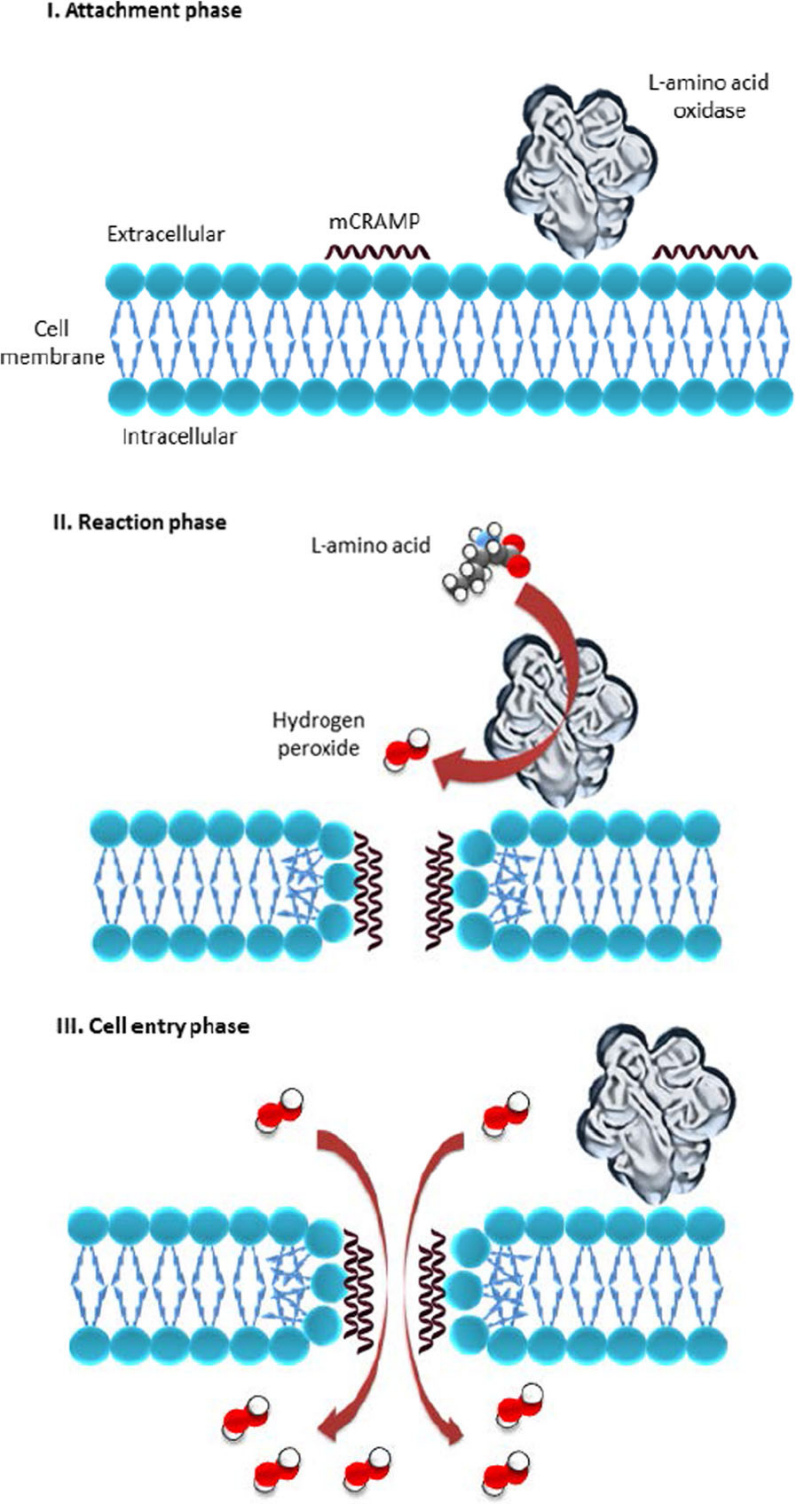
Proposed mechanism of synergistic antibacterial activity by combinational therapy of Oh-LAAO and mCRAMP. In the attachment phase, the treatment components, Oh-LAAO and mCRAMP, recognize and bind to the surface of MRSA. During the reaction phase, Oh-LAAO catalyses the oxidative deamination of l-amino acids into its corresponding keto acids with the release of hydrogen peroxide as a by-product, while mCRAMP reacts with the bacterial surface membrane leading to pore formation. Finally, in the entry phase the generated hydrogen peroxide enters the bacterial cells via mCRAMP-formed pores, leading to disruption of multiple intracellular component targets including proteins and DNA, subsequently causing more rapid cell lysis

The mechanisms of synergistic antimicrobial activities are not well understood. In general, synergistic activities are observed in antimicrobial drug combinations which act via different mechanisms and, in some cases, one treatment component involves a membrane targeting agent which often increases permeability [13]. It has been elucidated that the mechanism of mCRAMP antimicrobial activity involves binding on the bacterial surface to permeablize cells leading to loss of cytoplasmic content [23]. The enzymatic reaction of Oh-LAAO oxidizes l-amino acids to their corresponding keto acid with hydrogen peroxide released as a by-product; this has been proposed to be responsible for its bactericidal effects [3]. Additionally, Oh-LAAO has been demonstrated to bind to both Gram-positive and -negative bacterial surfaces, leading to high local concentrations of hydrogen peroxide which increases its antibacterial potency. The presence of mCRAMP which permeabilizes bacterial membranes could facilitate the entry of liberated hydrogen peroxide into the cytoplasm of pathogens leading to disruption of multiple intracellular component targets including proteins and DNA, subsequently causing more rapid cell lysis (Fig. 8).

AMPs are known to increase the permeability of bacterial membranes to synergize with conventional antibiotics [29]. Chimeric AMPs synthesized by combinations of functional groups from natural AMPs have demonstrated synergistic effects with conventional antibiotics including cefotaxime, ciprofloxacin, and erythromycin [30]. The synergistic effect observed in our study suggests that mCRAMP may contribute to boosting antibacterial activity by combinations with other antibiotics and antimicrobial proteins. Additionally, it is postulated that co-administrations with mCRAMP may not result in adverse effects related to drug-drug interactions or interference with antibacterial activity, considering it is an endogenous protein.

While some novel antibacterial proteins may perform well in vitro, problems with toxicity may limit their potential clinical application. In such instances, the compound may be salvaged by the use of an enhancer which synergizes with its effects, leading to a reduced dose required to achieve therapeutic efficacy and thereby preventing toxicity. This could be achieved with the use of a membrane permeabilizing AMP such as mCRAMP. It is therefore postulated that AMPs with this function may be used to reduce the administration dose of antibacterial proteins isolated from novel sources such as snake venoms which are highly efficacious towards pathogenic bacteria including resistant strains, but also exhibit high toxicity towards eukaryotic cells. LAAOs, phospholipase A2, and cathelicidins are included among the many promising antibacterial proteins that have been identified from snake venoms [31] which are efficacious against clinically important Gram-positive and Gram-negative bacteria. Therefore, co-administration with MSCs or mCRAMP may be a feasible solution to broadening the spectrum of antimicrobial agents available for the treatment of resistant bacteria.

## Conclusions

In conclusion, combinational therapy of MSCs and Oh-LAAO may be useful in the treatment of MRSA-infected cutaneous wounds as demonstrated by the mitigation of wound healing and synergistic antibacterial activity. Additionally, mCRAMP secretion by MSCs could also be explored for synergistic effects with conventional antibiotics and for reducing the dose of novel antimicrobial agents which exhibit high toxicity.

## Additional file

Additional file 1: Figure S1. Flow cytometry analysis of MSCs isolated from mouse adipose tissues at passage 3. The data obtained correspond to currently reported phenotype of MSCs which are positive for Sca-1, CD106, CD105, CD73, CD29, and CD44, while negative for CD45 and CD11b. **Figure S2.** Cellular morphology and staining of MSC differentiation assay. MSCs were incubated for 14 days for adipogenic differentiation and 21 days for chondrogenic and osteogenic differentiation. Differentiation of MSCs was confirmed with Oil Red O for adipocytes, Alizarin Red for osteocytes, and Safranin O for chondrocytes. The negative controls consisted of undifferentiated MSCs stained with either Oil Red O, Alizarin Red, or Safranin O in the corresponding assays. All images are of 10× magnification except for Oil Red O stained adipocytes and Safranin O stained chondrocytes which are of 20× magnification. Scale bars = 50 μm. **Figure S3.** SDS-PAGE performed on the expected Oh-LAAO fraction isolated from two-step Mono Q ion exchange chromatography and BioSep-Sec-S size exclusion chromatography. Standard 4% stacking gel and 12% resolving gel was used in the procedure. The results indicated the presence of a single protein band, denoting the protein has been purified to homogeneity. (DOCX 731 kb)

## Abbreviations

AMP: Antimicrobial peptide
AMR: Antimicrobial resistance
CaTx-II: *Crotalus adamanteus* toxin II
CFU: Colony-forming units
FAO: Fusidic acid ointment
H&E: Hematoxylin and eosin
LCMS: Liquid chromatography-mass spectrometry
MBC: Minimum bactericidal concentration
mCRAMP: Mouse cathelicidin-related antimicrobial peptide
MIC: Minimum inhibitory concentration
MRSA: Methicillin-resistant *Staphylococcus aureus*
MSC: Mesenchymal stromal cell
Oh-LAAO: *Ophiophagus hannah* l-amino acid oxidase, PBS, Phosphate-buffered saline
SDS-PAGE: Sodium dodecyl sulfate polyacrylamide gel electrophoresis
