# Supplementary material for: Synergistic antibacterial effect of co-administering adipose-derived mesenchymal stromal cells and Ophiophagus hannahl-amino acid oxidase in a mouse model of methicillin-resistant Staphylococcus aureus-infected wounds
Source: Stem Cell Res Ther. 2017 Jan 23;8:5. doi: 10.1186/s13287-016-0457-2 (PMC5259957; doi:10.1186/s13287-016-0457-2)

**Additional file 1**

**Synergistic antibacterial effect of co-administering adipose-derived mesenchymal stromal cells and *Ophiophagus hannah* L-amino acid oxidase in a mouse model of methicillin-resistant *Staphylococcus aureus* infected wounds.**

Mot Yee Yik^1^, Iekhsan Othman^1^, Sharifah Syed Hassan^1^*.

**Figure legends**

**Figure S1:** Flow cytometry analysis of MSCs isolated from mouse adipose tissues at passage 3. The data obtained corresponds to currently reported phenotype of MSCs which are positive for Sca-1, CD106, CD105, CD73, CD29, and CD 44, while negative for CD45 and CD11b.

**Figure S2:** Cellular morphology and staining of MSC differentiation assay. MSCs were incubated for 14 days for adipogenic differentiation and 21 days for chondrogenic and osteogenic differentiation. Differentiation of MSCs were confirmed with Oil Red O for adipocytes, Alizarin Red for osteocytes and Safranin O for chondrocytes. The negative controls consisted of undifferentiated MSCs stained with either Oil Red O, Alizarin Red or Safranin O in the corresponding assays. All images are of 10X magnification except for Oil Red O stained adipocytes and Safranin O stained chondrocytes which are of 20X magnification. Scale bars, 50µm.

**Figure** S**3:** SDS-PAGE performed on the expected Oh-LAAO fraction isolated from two-step Mono Q ion exchange chromatography and BioSep-Sec-S size exclusion chromatography. Standard 4% stacking gel and 12% resolving gel was used in the procedure. The results indicated the presence of a single protein band, denoting the protein has been purified to homogeneity.

**Figure S1**

**Figure S2**


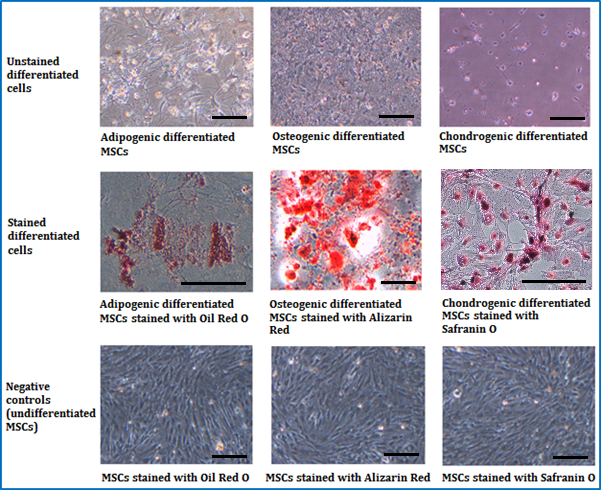


**Figure** S**3**


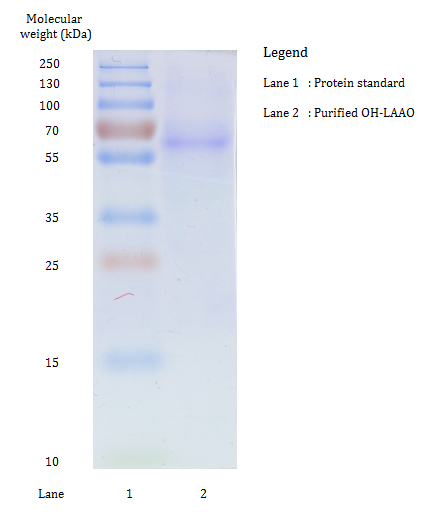

Supplement: Additional file 1: Figure S1. — Flow cytometry analysis of MSCs isolated from mouse adipose tissues at passage 3. The data obtained correspond to currently reported phenotype of MSCs which are positive for Sca-1, CD106, CD105, CD73, CD29, and CD44, while negative for CD45 and CD11b. Figure S2. Cellular morphology and staining of MSC differentiation assay. MSCs were incubated for 14 days for adipogenic differentiation and 21 days for chondrogenic and osteogenic differentiation. Differentiation of MSCs was confirmed with Oil Red O for adipocytes, Alizarin Red for osteocytes, and Safranin O for chondrocytes. The negative controls consisted of undifferentiated MSCs stained with either Oil Red O, Alizarin Red, or Safranin O in the corresponding assays. All images are of 10× magnification except for Oil Red O stained adipocytes and Safranin O stained chondrocytes which are of 20× magnification. Scale bars = 50 μm. Figure S3. SDS-PAGE performed on the expected Oh-LAAO fraction isolated from two-step Mono Q ion exchange chromatography and BioSep-Sec-S size exclusion chromatography. Standard 4% stacking gel and 12% resolving gel was used in the procedure. The results indicated the presence of a single protein band, denoting the protein has been purified to homogeneity. (DOCX 731 kb) [file 13287_2016_457_MOESM1_ESM.docx]
